# Arsenic, Cadmium, and Lead in Soils and Cereal Grains of the Pannonian Plain (Croatia): Soil-to-Grain Transfer and Dietary Exposure Assessment

**DOI:** 10.3390/foods15061036

**Published:** 2026-03-16

**Authors:** Danijel Brkić, Jelena Marinić, Dijana Tomić Linšak, Gordana Jurak, Dario Lasić, Jasna Bošnir, Dalibor Broznić

**Affiliations:** 1 Department of Environmental Hygiene, Teaching Institute of Public Health “Dr. Andrija Štampar”, Mirogojska cesta 16, 10000 Zagreb, Croatia; danijel.brkic@stampar.hr; 2 Independent Researcher, 51000 Rijeka, Croatia; jelena_marinic@hotmail.com; 3 Department for Health Ecology, Faculty of Medicine, University of Rijeka, Braće Branchetta 20, 51000 Rijeka, Croatia; dijanatl@uniri.hr; 4 Department for Scientific and Teaching Activity, Teaching Institute of Public Health County of Primorje-Gorski Kotar, Krešimirova 52a, 51000 Rijeka, Croatia; 5 Department of Analytical Techniques and Drug Control, Teaching Institute of Public Health “Dr. Andrija Štampar”, Mirogojska cesta 16, 10000 Zagreb, Croatia; gordana.jurak@stampar.hr; 6 Department for Food Safety and Quality, Teaching Institute of Public Health “Dr. Andrija Štampar”, Mirogojska cesta 16, 10000 Zagreb, Croatia; dario.lasic@stampar.hr; 7 Department of Environmental Health, Teaching Institute of Public Health “Dr. Andrija Štampar”, Mirogojska cesta 16, 10000 Zagreb, Croatia; 8 Environmental Health Engineering Study Program, University of Applied Health Sciences, Mlinarska cesta 38, 10000 Zagreb, Croatia; 9 Department of Medical Chemistry, Biochemistry and Clinical Chemistry, Faculty of Medicine, University of Rijeka, Braće Branchetta 20, 51000 Rijeka, Croatia; 10 Department of Environmental Health, Teaching Institute of Public Health of Primorje-Gorski Kotar County, Krešimirova 52a, 51000 Rijeka, Croatia

**Keywords:** heavy metals, arsenic, cadmium, lead, cereals, soil, transfer factor, exposure assessment, health risk, food safety

## Abstract

Heavy metals in agricultural systems pose a significant challenge to food security, especially in regions with long-term intensive land use. While the Pannonian Plain represents Croatia’s primary breadbasket, accounting for a significant portion of the nation’s cereal production, data on the soil-to-grain transfer of heavy metals and the associated human exposure risk are limited. The objective of this study was (i) to determine the concentrations of arsenic (As), cadmium (Cd), and lead (Pb) in agricultural soils and corresponding grains (wheat, barley, and maize) across four principal counties within the Pannonian region of Croatia; (ii) to evaluate the soil-to-grain transfer factors that varied regionally and among cereal types; and (iii) to assess the potential non-carcinogenic health risks for both adults and children highlighting differences in exposure due to body weight and consumption patterns. Soil and cereal grain samples were collected in 2019 and 2020, and metal concentrations were determined by ICP-MS after microwave acid digestion. The transfer of metals from soil to grain was estimated using the transfer factor (TF), while exposure assessment was conducted by calculating the estimated daily intake (EDI), hazard quotient (HQ), and hazard index (HI). Due to the nonlinear distribution of the data and the lack of strictly matched soil and grain samples, median metal concentrations pooled across all studied regions were used for exposure assessment. For As, a conservative approach was applied, assuming that 50% of the total As is in inorganic form. Additionally, a probabilistic risk assessment using Monte Carlo simulations was conducted to account for variability in body weight and cereal intake, providing a more comprehensive evaluation of potential exposure. The results showed differences in metal accumulation among cereal species, with wheat and barley tending to accumulate more Cd than maize, while As and Pb concentrations in grains were low for all crops studied. Although soil metal concentrations in Međimurje County were generally low, elevated TF values for As and Pb were observed, indicating enhanced soil-to-plant transfer under specific local soil conditions. In contrast, high soil metal concentrations in Slavonski Brod–Posavina County were associated with low TF values, suggesting limited bioavailability and restricted transfer to cereal grains. Both deterministic and probabilistic assessments indicated that the HQ and HI for adults and children were below 1, suggesting low non-carcinogenic risk from cereal consumption. These findings highlight pronounced regional and crop-specific differences in soil-to-plant metal transfer and confirm that low soil contamination does not necessarily imply low transfer potential, emphasizing the importance of integrated soil–plant–grain monitoring for food safety assessment.

## 1. Introduction

Potentially toxic metals, such as arsenic (As), cadmium (Cd), and lead (Pb), are among the most significant inorganic environmental contaminants due to their persistence, toxicity, and ability to accumulate in soil and living organisms [1,2,3]. Their presence in agricultural soils poses a long-term challenge for food security and public health, especially in regions with intensive agricultural production [2,4,5,6]. Soil plays a key role in metal cycling in agroecosystems, serving as the primary reservoir from which metals can be mobilized and taken up by plants [2,3,7]. The bioavailability of these metals in soil is influenced by several factors, including soil properties, pH, organic matter content, soil texture, and the presence of other chemical elements [3,5,6,7]. Although total metal concentrations in soil provide a basic indication of contamination levels, the actual risk to the food chain depends on their availability to plants and their transfer from soil to the edible parts of plants [6,7,8,9,10,11]. Once taken up by cereals, these metals can bioaccumulate along the soil–plant–human food chain, resulting in chronic dietary exposure. Inorganic As readily migrates from contaminated soil to cereals and is classified as a human carcinogen, linked to skin, lung, and bladder cancers, as well as cardiovascular and neurological disorders [12,13]. Cd tends to accumulate efficiently in cereal tissues, and long-term intake can impair renal function, cause bone demineralization (as observed in Itai-itai disease), and affect immune and respiratory health [12,13,14]. Pb is less mobile in cereals than Cd and As but can still accumulate in grains at worrying concentrations under contaminated conditions. Even low but chronic exposure is associated with neurotoxic effects, hematological changes, and cardiovascular and renal damage, particularly in children and other vulnerable populations [12,13].

Cereals such as wheat, barley, and maize form the basis of the global diet and are significant sources of carbohydrates, proteins, and micronutrients [6,8,9,10,11]. Due to their widespread distribution and daily consumption, cereals are one of the main routes of human exposure to metals through the diet [8,10,11,15,16]. Numerous studies indicate that As, Cd, and Pb levels in cereals can vary significantly depending on plant species, soil geochemical characteristics, and agricultural practices [5,6,8,9,10,11]. Globally, there is increasing attention to assessing the risks associated with dietary intake of metals, especially regarding long-term exposure to low concentrations [8,10,11,15,16,17]. Given these considerations, continuous evaluation of metal occurrence in cereals and associated exposure levels remains essential [1,17,18].

In the Republic of Croatia, particularly within the Pannonian Plain, agricultural soils have a long history of intensive cultivation and application of mineral fertilizers and pesticides, alongside environmental loading from industrial activities and traffic-related sources of pollution. Similar agroecosystems in Europe and Asia show that such activities contribute to elevated levels of As, Cd, and Pb in soils [3,4,5,19,20]. Previous research at the EU level has indicated spatial variability in metal content in soils, with some agricultural areas classified as requiring additional assessment and possible remediation measures [2]. However, data on the transfer of metals from soil to cereals and the consequent dietary exposure of the population in this part of Europe remain limited and fragmented [1,2,18].

Within the EU, monitoring of potentially toxic metals in soil and agricultural products is governed by a series of legislative and guidance documents aimed at ensuring food safety and protecting consumer health [1,2]. Despite a common regulatory framework, studies in different member states indicate significant regional differences in As, Cd, and Pb concentrations in soils and cereals, attributed to diverse geological substrates, historical pollution, and the intensity of agricultural production [2,4,5,19]. The differences between Western and Eastern Europe are particularly pronounced, with elevated levels of Cd in soils and cereals recorded in some areas, associated with the long-term use of phosphate fertilizers [2,3,5,20].

In Southeastern Europe and the Balkans, available data on metal content in agricultural soils and cereals remain relatively limited and unevenly distributed [2,21,22]. Previous research from neighboring countries indicates locally elevated concentrations of As, Cd, and Pb in soils, particularly in areas with significant geogenic influence, a history of mining, or proximity to industrial sources [21,23,24]. For example, agricultural soils in northeastern Romania and areas near metallurgical and steel plants have been found to contain locally elevated concentrations of a range of metals (including Cr, Ni, Cu, As, Pb, Hg and Cd) that exceed national threshold values for sensitive land use [21,22]. Studies in Serbia and the wider Balkans also indicate the long-term presence of elevated concentrations of Pb and other metals related to mining and smelting activities [23,24]. However, results on metal transfer to cereals and other crops are often heterogeneous, and estimates of dietary exposure are rarely conducted systematically or comparably across regions [21,25,26,27]. Recent studies in Romania and Atlantic-European peripheral areas show that even with a relatively small number of cereals or plant productions, assessments of metal intake can indicate potential health risk, but long-term, standardized monitoring programs are lacking [25,26,28]. In this context, research conducted in the Republic of Croatia, located at the crossroads of Central Europe and the Balkans, can provide valuable insights into the behavior of metals in agroecosystems in this part of Europe and allow for comparison with data from other EU Member States and Southeastern European countries [2].

The relationship between soil and plant metal concentrations is often assessed using the transfer factor (TF), which provides a quantitative measure of the efficiency of metal transfer from soil to plant [8,9,10,11,29]. Although TF is a simplified indicator, its use in regional studies enables comparison of different crops and environmental conditions and contributes to a better understanding of metal behavior in agroecosystems [6,8,9,10,29]. In addition to evaluating metal transport, there is increasing emphasis on assessing human dietary exposure and associated health risks, most commonly through the calculation of estimated daily intake (EDI), hazard quotient (HQ), and overall hazard index (HI) [8,10,11,15,16,17,18]. This integrated approach allows for the consideration of potential risks under real-world dietary conditions, which is crucial for developing scientifically based recommendations and risk management measures [11,15,16,17,18].

Despite numerous international studies on the presence of potentially toxic metals in soils and cereals, integrated assessments that simultaneously cover soil contamination, metal transfer from soil to grain, and dietary exposure assessment are still limited for the region of South-Eastern Europe. In the Republic of Croatia, especially in the Pannonian Plain, as one of the country’s most important agricultural areas, data on the related analysis of metal concentrations in soil and cereals and their implications for the health risk to the population remain fragmented. This study provides a comprehensive regional assessment of As, Cd, and Pb in soils and major cereals of the Pannonian Plain, with the calculation of transfer factors and the assessment of non-carcinogenic risk. By linking environmental, agronomic, and public health components, the work contributes to understanding metal dynamics in the soil–plant–human system within an important agroecosystem of Central and South-Eastern Europe. Given this context, the aims of this study were (i) to determine the concentrations of As, Cd, and Pb in soils and cereal grains (wheat, barley, and maize) from the Pannonian Plain of the Republic of Croatia; (ii) to estimate the transfer of metals from soil to grain using the transfer factor; and (iii) to assess the exposure and non-carcinogenic health risk for different population, including sensitive subgroups through cereal grain consumption.

## 2. Materials and Methods

### 2.1. Research Area

The research was conducted in the Republic of Croatia in the Pannonian Plain, which is one of the most important agricultural areas in the country, especially for cereal cultivation. The area is bounded by the Mura River in the northwest (46°50′ N, 16°10′ E), the Sava River in the south (45°50′ N, 16°30′ E), the Drava River in the north (46°40′ N, 17°30′ E) and the Danube River in the east (45°50′ N, 18°50′ E). The total area of the Pannonian Plain in Croatia is approximately 31,100 km^2^, which makes up more than half of the country’s territory, and is home to about two-thirds of the total population [30]. The relief of the area mainly consists of lowland basins, gently undulating surfaces and isolated hills, with an average altitude of approximately 200 m [30,31]. The climate of the Pannonian Plain is moderately warm and humid. Average January temperatures range between –2 and 0 °C, while average July temperatures range from 22 to 24 °C. Annual precipitation varies from approximately 780 mm in the eastern part to approximately 900 mm in the west, while the average duration of sunshine hours is between 1750 and 2000 h per year [32]. Climatic conditions during the study period (2019–2020) were typical for the observed area. In 2019, average January temperatures ranged from –1 to 1 °C, and July temperatures from 21 to 23 °C, with a total annual precipitation of approximately 900 mm and an average duration of sunshine hours of approximately 1750 h. In 2020, average January temperatures ranged from –2 to 0 °C, while average July temperatures ranged from 22 to 24 °C. Total annual precipitation was about 850 mm, and the duration of sunshine hours was approximately 1800 h [33,34].

The Pannonian Plain is a key area for cereal production in Croatia. During the observed period, high yields of wheat (*Triticum aestivum* L.), barley (*Hordeum vulgare* L.) and maize (*Zea mays* L.) were achieved, with approximately 781,000 t of wheat, 263,000 t of barley and 2.2 million t of maize produced in 2019, while in 2020, production increased to approximately 842,000 t of wheat, 311,000 t of barley and 2.4 million t of maize [35]. Given the importance of these cereals in the diet of the population and their widespread presence in everyday consumption, this area was selected for conducting research with the aim of assessing the potential intake and transfer of heavy metals through the food chain.

### 2.2. Selection of Counties and Sampling Locations

**Figure 1:**
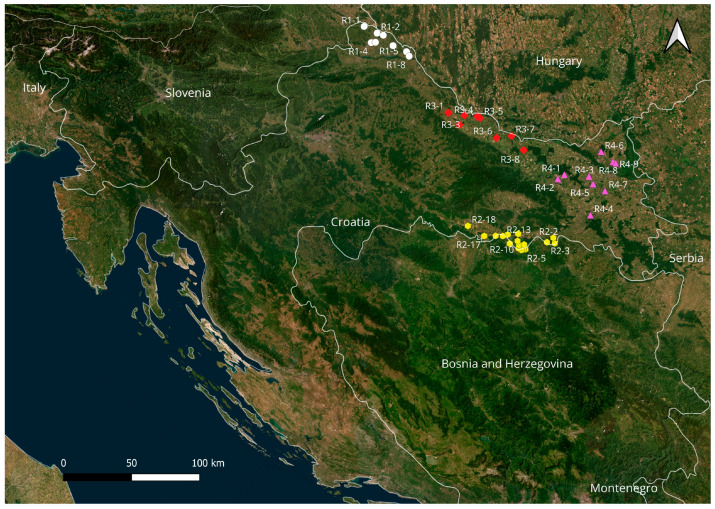
Geographical representation of cereal sampling locations in selected counties of the Pannonian Plain and the Republic of Croatia. R1 = Međimurje County, R2 = Slavonski Brod–Posavina County, R3 = Virovitica–Podravina County, R4 = Osijek–Baranja County.

The research was conducted in four counties in the northern and eastern part of the Republic of Croatia: Međimurje (R1), Slavonski Brod–Posavina (R2), Virovitica–Podravina (R3) and Osijek–Baranja (R4). The selected counties are located within the Pannonian Plain and encompass spatially and agriculturally heterogeneous areas with intensive crop production. Međimurje County is located in the northernmost part of Croatia, between the Mura and Drava rivers (R1; 46°19′–46°30′ N; 16°17′–16°48′ E). Slavonski Brod–Posavina County is located in the eastern part of the country along the Sava River (R2; 45°03′–45°14′ N; 17°16′–18°10′ E). Virovitica–Podravina County is located along the Drava River in the northeastern part of Croatia (R3; 45°35′–45°57′ N; 17°09′–17°53′ E), while Osijek–Baranja County is located in eastern Croatia between the Drava and Danube rivers (R4; 45°17′–45°47′ N; 18°12′–18°48′ E). The geographical location and distribution of the sampled locations are shown in Figure 1.

Sampling locations were selected within agricultural areas intended for cereal cultivation, with the aim of capturing spatial variability within each county. Soil samples were collected from crop plots, while cereal samples were collected from agricultural farms during the growing season and harvest, within the same administrative areas.

Soil and grain samples were collected within the same counties, but they do not represent strictly paired samples, i.e., they were not sampled from identical sampling locations. Although both types of samples are spatially linked to the same regional areas, they constitute independent data sets. This approach enables the assessment of regional patterns of metal distribution in soil and their accumulation in cereal grains but does not allow soil–plant comparisons at the individual field level. Due to the uneven number of soil and grain samples across counties and the absence of paired sampling, metal concentrations in soil and grains are described using median values, which reduce the influence of extreme values and provide representative regional estimates. This methodological framework forms the basis for the application of non-parametric statistical analyses and for calculating regional soil-to-grain transfer factors (TF).

### 2.3. Soil Sampling

Soil samples were collected during 2019 and 2020 from agricultural areas in four counties (R1–R4) representing the dominant pedological and agroecological conditions within each county. Sampling was carried out during the harvest period of the respective cereal crops, corresponding to mid-June and the first half of July for wheat and barley; and mid-September to October for maize, wheat and barley; and mid-September to October for maize. Soil sampling was carried out manually using a stainless-steel probe and shovel, which were thoroughly cleaned and rinsed with distilled water before each use to prevent possible cross-contamination of samples. Samples were collected from the arable soil layer (0–30 cm), which represents the zone of greatest interaction between the soil and the plant root system and the primary reservoir of metals potentially available to plants. A composite soil sample was formed on each agricultural plot, consisting of 5 to 10 individual subsamples collected according to a “W”-shaped sampling pattern, in accordance with ISO 18400-104:2018 [36]. This approach enabled the reduction in spatial heterogeneity of the soil within the plot and ensured the representativeness of the sample for further analyses.

A total of 43 soil samples were collected, including 8 samples from Međimurje County, 18 samples from Slavonski Brod–Posavina County, 8 samples from Virovitica–Podravina County, and 9 samples from Osijek–Baranja County. The number of samples per county was adjusted to the availability of agricultural areas and the spatial heterogeneity of the soil within each region. After sampling, the soil samples were stored in clean polyethylene bags, labeled, and transported to the laboratory. In the laboratory, the samples were air-dried at room temperature, ground, and sieved through a 2 mm sieve to remove larger fragments and ensure sample homogeneity. The prepared samples were stored in closed containers until chemical analysis. Soil samples were collected at the county level and were not intended for direct pairing with individual cereal samples.

### 2.4. Cereal Sampling

Cereal samples (wheat, barley, and maize) were collected during the harvest in 2019 and 2020 in the area of four counties of the Pannonian Plain (R1–R4). A random “W”-shaped sampling pattern was applied on each agricultural plot in accordance with Commission Regulation (EC) No 333/2007 [37]. Multiple individual plant units (wheat and barley ears, maize cobs) were collected along a predefined sampling path and combined into a composite sample. Each composite sample was then quartered to obtain a representative subsample of approximately 2 kg, packed in clean plastic bags, and transported to the laboratory in a refrigerated container (+4 °C). In the laboratory, the samples were dried to constant mass, then ground and homogenized.

A total of 196 cereal samples were collected over two seasons (2019: 115; 2020: 81). The highest number of samples was collected in Slavonski Brod–Posavina, Virovitica–Podravina and Osijek–Baranja counties, while the lowest number of samples was collected in Međimurje County. All three cereal types were represented in all counties, with the proportions of individual crops varying between counties and seasons. Cereal samples were collected independently of soil sampling sites within the same counties and study period. The distribution of collected samples by counties and cereal types is shown in Table 1.

### 2.5. Sample Preparation

#### 2.5.1. Preparation of Cereal Samples

Before chemical analysis, grain samples were cleaned of surface impurities and washed with distilled water to remove external contaminants. After that, initial drying was carried out in a Memmert UN 260 dryer (Memmert GmbH, Schwabach, Germany) at 105 °C for 30 min to remove surface and free water. Drying then continued at 75 °C until a constant mass was reached. The dried samples were mechanically pulverized and homogenized using a Retsch Cyclone Mill (Retsch GmbH, Haan, Germany) and then sieved through a 2 mm sieve to ensure a uniform particle size structure and sample representativeness for further analysis. All procedures were performed using titanium knives and glass laboratory glassware previously cleaned with 1 M HNO_3_ (Scharlau, Sentmenat, Spain) and ultrapure water (Merck, Darmstadt, Germany), to prevent cross-contamination. The ground samples were stored in sterile PVC containers, hermetically sealed and clearly labeled, and kept until microwave acid digestion and instrumental analysis.

#### 2.5.2. Preparation of Soil Samples

After collection, soil samples were air-dried at room temperature, without the application of elevated temperatures to prevent changes in chemical composition. The dried soil samples were mechanically crushed and sieved through a 2 mm sieve to remove stones, plant debris, and other inhomogeneous fractions. The sieved soil samples were further homogenized to ensure the representativeness of the subsample for analysis. All procedures were carried out using inert tools and laboratory dishes previously cleaned with 1 M HNO_3_ and ultrapure water in order to prevent metal contamination. Prepared soil samples were stored in clean, closed polyethylene containers and kept until microwave digestion and elemental analysis.

### 2.6. Microwave Acid Digestion of Samples

To determine the concentrations of As, Cd, and Pb in soil and grain samples, microwave acid digestion was performed. Approximately 0.5–1.0 g of the homogenized sample was transferred to quartz digestion vessels. A 3 mL of 67% HNO_3_ and 1 mL of 30% H_2_O_2_ (Alkaloid, Skopje, North Macedonia) were added to the sample, for more complete oxidation of organic matter, especially in plant samples. Digestion was performed in an MLS-1200 Mega microwave system (Milestone, Sorisole, Italy) following the manufacturer’s recommended procedures for food and soil matrices [38]. A customized program: 30 min of heating from 0 W to 1500 W, 10 min of power maintenance at 1500 W, and 30 min of cooling to 0 W. The maximum temperature reached during the procedure was 220 °C, and the maximum pressure was 120 bar. After digestion, the solutions were cooled to room temperature, quantitatively transferred to 50 mL volumetric flasks and made up with ultrapure water. Metal concentrations in the resulting solutions were determined by ICP-MS.

### 2.7. ICP-MS Analysis

As, Cd and Pb concentrations in digested soil and cereal grain samples were determined by inductively coupled plasma-mass spectrometry (ICP-MS) using an Agilent 7900 system (Agilent Technologies, Santa Clara, CA, USA). The instrument is optimized for high sensitivity and signal stability, with minimal formation of oxides and doubly charged ions. ICP-MS analysis parameters included a plasma gas flow (argon) of 15 L/min, auxiliary gas 0.9 L/min, nebulizer gas flow 1.07 L/min, radio frequency (RF) power of 1180 W, and peristaltic pump sample flow 0.4 mL/min. A Scott-type cooled spray chamber was used to ensure plasma stability and measurement repeatability, and the quadrupole analyzer was operated in standard mode and in collision/reaction cell mode with helium (4.3 mL/min) in KED mode to reduce polyatomic interference.

The quantification of the elements was performed using the method of time-resolved signal acquisition (dwell time 50 ms), with three repetitions per sample and a total integration time of 1000 ms. Rhodium (Rh, 10 µg/L) was used as an internal standard to correct for instrumental variations and matrix effects. The electronic multiplier detector worked in pulse-counting mode, and the mass resolution was set to the unit mass for efficient separation of analytes from isobaric interferences. The peak hopping method of measurement was used for all three repetitions per sample. The isotopes chosen for quantification were ^75^As, ^111^Cd, and ^208^Pb, due to their high natural abundance and minimal interference in the KED mode. The complete list of analyzed isotopes, associated masses, internal standards, and applied collision-reaction cell corrections is shown in Table 2.

### 2.8. Quality Control and Method Validation (QA/QC)

Quality control and validation of the analytical method were carried out in accordance with current recommendations for the determination of elements in agricultural and environmental samples and according to the guidelines of HRN EN ISO/IMEC 17025:2017 [39,40]. Quantification of elements was performed using certified multi-element standard solutions in acidic medium (CPA Chem, Sofia, Bulgaria). Quality control procedures were applied equally to grain and soil samples.

In each analytical series, procedural blanks and blind samples were analyzed in parallel with the actual samples in order to check for potential contamination during sample preparation and the stability of the instrumental response. Procedural blanks underwent all preparation and digestion steps identically to the actual samples.

The linearity of the calibration curves for elements was confirmed through seven concentration levels. The calibration range was 0.2–200 µg/L for As and Pb and 0.05–50 µg/L for Cd. All calibration graphs showed regression coefficients *R*^2^ ≥ 0.999, which indicates excellent linearity of the method in the examined concentration range.

The limits of detection (LOD) and quantification (LOQ) were calculated according to the following equations:

(1) LOD=(3.3σ)/S

(2) LOQ=(10σ)/S

In Equations (1) and (2), σ represents the standard deviation of the signal of procedural blanks (n = 3), and S is the slope of the calibration curve. LOD and LOQ are expressed in mg/kg dry weight of the sample, taking into account the weight of the sample and the final volume of the digestate. The obtained LOD and LOQ values for the analyzed elements in cereal and soil samples are shown in Table 3.

The accuracy of the grain analysis method was verified by analyzing the certified reference material NIST SRM 1567b (Wheat Flour; Sigma-Aldrich Chemie GmbH, Munich, Germany), which was included after every tenth sample in the analytical run. The recovery percentages for As, Cd, and Pb ranged between 93 and 107%, which is in accordance with the acceptable criteria for the analysis of metals in food matrices according to EU guidelines [37].

Where matrix-matched certified reference materials were unavailable for soil, the accuracy of the method was verified by analyzing matrix-doped samples (spike recovery), control standard solutions, and repeated measurements of representative samples. The recovery percentages for As, Cd, and Pb in the soil samples were in the 90–110% range. The precision of the method was estimated from the relative standard deviation (RSD), calculated from the analysis of three independently prepared subsamples for each matrix (cereals and soil), where each subsample was analyzed in duplicate. All calculated RSDs were less than 10%, and the highest recorded value was 9%, confirming good repeatability of the procedure, homogeneity of samples and stability of the instrumental system. The use of an internal standard (rhodium, Rh, 10 µg/L), matrix-matched calibrations and optimized operation of the ICP-MS system further ensured control of instrumental drift and reduction in matrix effects. All metal concentrations in grains and soil are expressed in mg/kg dry weight.

### 2.9. Regulatory Guideline Values/Reference Limits

To assess the potential relevance of the measured metal concentrations from an environmental and food safety perspective, regulatory guidelines for agricultural soils and maximum permitted levels of metals in cereals were compiled based on relevant European legislation and scientific assessments (Table 4). The guidelines for soils are based on commonly applied ranges in EU Member States and EFSA assessments, while the maximum permitted levels of metals in cereals are taken from Commission Regulation (EU) 2023/915 [41]. Since maximum permitted levels for metals in cereals are expressed on a wet weight basis, grain concentrations were converted from dry weight to wet weight, assuming an average moisture content of 14% to enable comparison with regulatory limits. These reference values were used as a basis for comparing measured concentrations in soil and grains and for discussing the potential risks associated with metal accumulation in the study counties.

### 2.10. Transfer Factor (TF)

The transfer factor (TF) was used to estimate the transfer of metals from soil to the above-ground parts of plants, i.e., the edible part of cereals (grain). TF is defined as the ratio of the metal concentration in the plant to the metal concentration in the soil and is often used as an indicator of the bioavailability of metals and their accumulation potential in plants [42,43]. TF was calculated according to the following equation:

(3) $$\mathrm{TF}=\frac{C_{\left(\mathrm{plant}\right)}}{C_{\left(\mathrm{soil}\right)}}$$

In Equation (3), *C*_(plant)_ and *C*_(soil)_ represent metal concentration in the cereal grain and in the soil (mg/kg dry weight).

Because soil and cereal samples were not spatially paired, TFs were calculated using county-level median concentrations of metals in soils and cereals. The resulting TF values represent regional transfer characteristics and are interpreted descriptively. TF values < 1 reflect limited transfer, values ≈ 1 indicate moderate transfer, and values > 1 suggest increased metal accumulation in the plant relative to the soil. Elevated TF values may indicate higher metal bioavailability in the soil, specific physiological mechanisms of uptake and transfer in the plant, as well as the influence of soil properties, including chemical soil properties (pH, organic matter content) and physical/pedological properties (soil texture) [14,42,43].

The calculation of the TF is based on median metal concentrations and does not take into account spatial soil heterogeneity, variability within agricultural areas, or the influence of specific agronomic practices. Furthermore, because soil and cereal samples were not strictly paired, these results provide a regional exposure assessment rather than a direct correlation between specific soil sampling locations and corresponding cereal samples.

### 2.11. Exposure and Health Risk Assessment

Exposure and potential non-carcinogenic health risk assessment were conducted for both adult and child populations for As, Cd, and Pb, based on metal concentrations determined in cereal grain samples (barley, wheat, and maize). Median metal concentrations in cereal grains, pooled for all studied regions, were used for exposure assessment calculations. The use of median is justified by the asymmetric distribution of data and the presence of extreme values, which reduces the influence of outliers and provides a more robust and conservative estimate of typical dietary metal intake. Metal concentrations used for exposure assessment were expressed on a dry weight basis. For comparison with regulatory maximum levels, which are expressed on a wet weight basis, grain concentrations were converted assuming an average moisture content of 14%. However, all exposure metrics (EDI, HQ, and HI) were calculated using dry weight concentrations, representing a conservative exposure scenario.

#### 2.11.1. Estimated Daily Intake (EDI) and Non-Carcinogenic Risk Assessment (HQ and HI)

The Estimated Daily Intake (EDI) of metals from cereal grain consumption was calculated according to the following equation:

(4) $$\mathrm{EDI}=\frac{C\times \mathrm{IR}}{\mathrm{BW}}$$

In Equation (4), *C* is the median metal concentration in the edible portion of cereal grains (mg/kg), IR is daily cereal intake (kg/day), and BW is average adult body weight (kg). In accordance with EFSA Scientific Committee guidance, a default body weight of 70 kg is used for the European adult population (≥18 years) [44]. For the estimation of dietary metal intake, assumed average daily consumption values of 150 g of wheat, 10 g of barley, and 30 g of maize per person were used, as indicative values consistent with cereal consumption data reported for Croatian adults [45,46]. In addition to the adult scenario, exposure was also assessed for children. For this population group, a body weight of 20 kg was assumed. Daily cereal consumption values were set at 60% of the adult intake, in order to approximate typical dietary patterns of children while maintaining proportional consistency with the adult exposure scenario. The same calculation procedure was applied to both population groups to allow direct comparison of the results. EDI values are expressed in mg/kg body weight per day.

The non-carcinogenic risk for each metal was estimated using the hazard quotient (HQ) calculated as

(5) $$\mathrm{HQ}=\frac{\mathrm{EDI}}{\mathrm{Reference}\;\mathrm{Value}}$$

The overall non-carcinogenic health risk was estimated by calculating the Hazard Index (HI), defined as the sum of the individual hazard quotients:

(6) $$\mathrm{HI}={\mathrm{HQ}}_{\mathrm{As}}+{\mathrm{HQ}}_{\mathrm{Cd}}+{\mathrm{HQ}}_{\mathrm{Pb}}$$

The HI values were used to provide an indicative estimate of the cumulative risk of metal exposure through cereal grain consumption.

#### 2.11.2. Reference Values and Metal-Specific Assumptions

Since total As was determined in the samples and chemical speciation was not performed, for health risk assessment, it was assumed that 50% of the total As is inorganic arsenic. This assumption represents a conservative approach commonly used in dietary risk assessments when speciation data are not available [47,48]. The following reference value for the Tolerable Daily Intake (TDI) was used [47]:

TDI_As_ = 0.0003 mg/(kg day)

The HQ for As was calculated according to the formula

(7) $${\mathrm{HQ}}_{\mathrm{As}}=\frac{\mathrm{EDI}}{{\mathrm{TDI}}_{\mathrm{As}}}$$

The TDI value for Cd was taken from the recommendations of the European Food Safety Authority (EFSA) and was derived from the tolerable weekly intake (TWI) of 2.5 µg/kg body weight, converted to a daily basis [49]

TDI_Cd_ = 0.00036 mg/(kg day)

The HQ for Cd was calculated as

(8) $${\mathrm{HQ}}_{\mathrm{Cd}}=\frac{\mathrm{EDI}}{{\mathrm{TDI}}_{\mathrm{Cd}}}$$

No tolerable daily or weekly intake has been established for Pb, as no safe exposure threshold has been identified. Therefore, the risk assessment was carried out using the benchmark dose lower confidence limit (BMDL) approach, in accordance with EFSA recommendations [50]. For both adult and child populations, the following value was applied [50]:

BMDL_Pb_ = 0.0015 mg/(kg day)

The HQ for Pb was calculated as

$${\mathrm{HQ}}_{\mathrm{Pb}}=\frac{\mathrm{EDI}}{{\mathrm{BMDL}}_{\mathrm{Pb}}}$$

The same toxicological reference values (TDI and BMDL) were applied for both adult and child populations, as no age-specific threshold values have been established for the assessed metals. The exposure and health risk assessment is based on median metal concentrations in cereals and does not take into account possible differences in metal bioavailability, food preparation methods, or individual dietary habits. Furthermore, because soil and cereal samples were not strictly paired, these results provide a regional exposure assessment rather than a direct correlation between specific soil sampling locations and corresponding cereal samples.

### 2.12. Statistical Analysis

Statistical data processing was performed using the commercial software Statistica^®^ v.14 (TIBCO Software Inc., Palo Alto, CA, USA), with the statistical significance level set at *p* < 0.05.

Due to the uneven number of samples across counties, the absence of paired soil-cereal samples, and the pronounced variability of metal concentrations, nonparametric statistical methods were applied. Elemental concentrations are expressed as medians with range values, while means and standard deviations are provided for descriptive purposes only. Differences in metal concentrations between counties within a single cereal species, as well as differences between cereal species, were examined using the Kruskal–Wallis rank test. When statistically significant differences were detected, post hoc multiple comparisons of pairs of groups were performed with correction for the level of significance. The results of statistical tests are presented using mean ranks, H values, and corresponding *p*-values. Different letter designations used in tables and graphs indicate statistically significant differences between groups (*p* < 0.05).

To assess the transfer of metals from soil to plants, the transfer factor (TF) was calculated. Since soil and cereal samples were not spatially matched, TF values were interpreted descriptively, without conducting statistical significance tests. To reduce the impact of extreme values, the population’s exposure to metals through cereal consumption was assessed using median metal concentrations for each cereal type, aggregated at the county level. The estimated daily intake (EDI), hazard quotient (HQ), and hazard index (HI) were calculated for both adult and child populations using a deterministic approach, and the resulting risk indicators were interpreted without additional statistical inference, since they are based on unique representative values.

In addition to the deterministic approach, a probabilistic risk assessment was performed using Monte Carlo simulation to take into account the variability of body weight and daily cereal consumption rates. The input parameters of the model were assigned appropriate probability distributions, and 10,000 iterations were performed to calculate the distributions of HQ and HI values for the adult and child populations. This approach allows for an assessment of the range of possible values of the risk indicators and the probability of exceeding the reference value (HQ or HI > 1), which provides a more realistic representation of exposure compared to a deterministic model based on a single representative value. The simulation results are presented in the form of probability distributions and characteristic percentiles (P5, P50, and P95), which allow for the assessment of typical, as well as elevated, exposure levels within the observed population. Monte Carlo simulations were performed in the Wolfram Mathematica^®^ software package, version 12 (Wolfram Research, Inc., Champaign, IL, USA).

## 3. Results

### 3.1. Metal Concentrations in Soils

The concentrations of As, Cd, and Pb in the soils of the studied counties are presented in Table 5 and show pronounced spatial variability among regions, as well as varying degrees of internal variability within individual counties. The lowest concentrations of all three metals were recorded in Međimurje, where the average values were 0.050 ± 0.015 mg/kg for As, 0.222 ± 0.058 mg/kg for Cd, and 0.154 ± 0.037 mg/kg for Pb. The median values were close to the mean values, confirming relatively uniform distribution within the data set, while the CV (24–30%) indicates moderate sample heterogeneity within the county. The highest soil metal concentrations were determined in the Slavonski Brod–Posavina County, especially for As and Pb. The average concentrations were 11.811 ± 4.991 mg/kg for As, 0.368 ± 0.172 mg/kg for Cd, and 31.911 ± 7.735 mg/kg for Pb. A wide range between minimum and maximum metal concentrations was recorded, particularly for Pb (21.60–50.16 mg/kg), indicating significant spatial variability within the county. Furthermore, moderate CVs (24–47%) confirm a heterogeneous spatial distribution of metals across the study area. Moderate metal concentrations were recorded in the Virovitica–Podravina County, with average values of 0.499 ± 0.075 mg/kg for As, 0.011 ± 0.004 mg/kg for Cd, and 1.669 ± 0.599 mg/kg for Pb. Compared to other counties, this county shows the lowest degree of variability, especially for As (CV = 15%), indicating a relatively homogeneous distribution of metals in soils, while Cd and Pb showed somewhat higher variability (CV = 31% and 36%, respectively). Soils of Osijek–Baranja County were characterized by elevated metal concentrations with pronounced internal variability. Average concentrations were 3.362 ± 1.557 mg/kg for As, 0.103 ± 0.035 mg/kg for Cd, and 11.224 ± 5.041 mg/kg for Pb. The CV (33–46%) indicates an uneven spatial distribution of metals within the county.

Analysis of differences in metal concentrations among counties showed statistically significant differences for all three elements. A significant difference was found for As (H(3, N = 43) = 35.83, *p* < 0.001), with the highest concentrations recorded in the Slavonski Brod–Posavina County and the lowest in Međimurje. A similar pattern was observed for Pb (H(3, N = 43) = 37.68, *p* < 0.001), while a different pattern was found for Cd (H(3, N = 43) = 30.40, *p* < 0.001). Post hoc analysis showed that As and Pb concentrations in Međimurje were statistically significantly lower than in the Slavonski Brod–Posavina and Osijek–Baranja counties (*p* < 0.05), while the Virovitica–Podravina showed intermediate values with partial overlap of statistical groups. In contrast, for Cd, Virovitica–Podravina had significantly lower concentrations than all other counties (*p* < 0.01).

### 3.2. Metal Concentrations in Cereals

#### 3.2.1. Barley (*Hordeum vulgare* L.)

As, Cd and Pb concentrations in barley grains showed marked spatial variability among the observed counties (Table 6). The mean values of As concentrations in barley ranged from 0.007 ± 0.002 mg/kg in Osijek–Baranja County to 0.030 ± 0.022 mg/kg in Slavonski Brod–Posavina County. Median values followed the same trend, with the highest median concentration recorded in Slavonski Brod–Posavina County (0.025 mg/kg), while lower values were found in Međimurje (0.009 mg/kg) and Osijek–Baranja County (0.007 mg/kg). The CV indicates a high heterogeneity of concentrations, especially in Međimurje County (CV = 100.9%). The average Cd concentrations varied between 0.018 ± 0.008 mg/kg in Virovitica–Podravina County and 0.049 ± 0.022 mg/kg in Slavonski Brod–Posavina County, with medians ranging from 0.017 to 0.046 mg/kg. Cd variability was moderate to high (CV = 27.3–45.8%). Pb concentrations in barley showed a similar pattern, with the highest mean values in Slavonski Brod–Posavina County (0.051 ± 0.034 mg/kg), while lower concentrations were recorded in Međimurje (0.022 ± 0.017 mg/kg), Virovitica–Podravina (0.026 ± 0.016 mg/kg) and Osijek–Baranja County (0.028 ± 0.005 mg/kg), and CV showed high heterogeneity (17.6–78.8%).

Statistical analysis confirmed significant differences in metal concentrations among counties. Kruskal–Wallis test showed statistically significant differences for As (H = 12.80, *p* = 0.005), Cd (H = 20.63, *p* < 0.001), and Pb (H = 8.58, *p* = 0.035). Post hoc analysis of multiple comparisons indicated that the concentration of As in barley from Slavonski Brod–Posavina County was statistically significantly higher compared to Osijek–Baranja County, while the differences among the other counties were insignificant. For Cd, the concentration of Cd in Slavonski Brod–Posavina County was significantly higher compared to Virovitica–Podravina County, while the differences among the other counties were not statistically significant. For Pb, significantly lower concentrations were recorded in Međimurje County compared to Slavonski Brod–Posavina County, while the differences among the other counties were insignificant.

#### 3.2.2. Wheat (*Triticum aestivum* L.)

Significant spatial variability in As, Cd, and Pb concentrations was observed in wheat across the surveyed counties, and the results are presented in Table 7. Mean As concentrations ranged from 0.007 ± 0.003 mg/kg in Međimurje County to 0.025 ± 0.023 mg/kg in Slavonski Brod–Posavina County, with median values following a similar trend (0.007–0.017 mg/kg). Variability within counties was high (CV = 41.7–108.99%). For Cd, mean values ranged from 0.030 ± 0.013 mg/kg in Međimurje to 0.050 ± 0.028 mg/kg in Slavonski Brod–Posavina County, while medians showed a smaller interregional difference. Cd variability was moderate (CV = 41–56%). Pb concentrations in wheat showed a similar pattern, with the lowest mean values in Međimurje (0.015 ± 0.009 mg/kg), while slightly higher values were found in Slavonski Brod–Posavina (0.030 ± 0.028 mg/kg) and Virovitica–Podravina County (0.024 ± 0.016 mg/kg). CV values (41–91.87%) indicate a heterogeneous distribution of Pb within counties.

Statistical analysis confirmed significant differences in As concentrations among counties (H = 12.89; *p* < 0.01), with Slavonski Brod–Posavina County having significantly higher As concentrations than Međimurje and Osijek–Baranja counties. Differences in Cd and Pb concentrations among counties were not statistically significant (*p* > 0.05).

#### 3.2.3. Maize (*Zea mays* L.)

In maize, spatial variability of As, Cd, and Pb concentrations is shown in Table 8. Average As concentrations in soils ranged from 0.011 ± 0.007 mg/kg in Virovitica–Podravina County to 0.019 ± 0.011 mg/kg in Međimurje County, while median values were uniform (0.013–0.022 mg/kg). Variability within regions was high (CV = 55.9–70.3%). The Kruskal–Wallis test showed a marginally significant effect of county on As concentrations (H = 7.92; *p* = 0.048), but post hoc analysis did not reveal statistically significant differences between individual pairs of counties. Average Cd concentrations varied from 0.006 ± 0.003 mg/kg in Međimurje to 0.009 ± 0.007 mg/kg in Osijek–Baranja County, while medians showed the lowest values in Međimurje and Slavonski Brod–Posavina (0.005–0.006 mg/kg) and the highest in Osijek–Baranja (0.008 mg/kg). Variability within regions was extremely high, especially in Slavonski Brod–Posavina County (CV = 98.0%). Statistical analysis confirmed significant differences in Cd concentrations among counties (H = 16.52; *p* = 0.0009), with concentrations in Osijek–Baranja being significantly higher than in Međimurje and Slavonski Brod–Posavina (*p* < 0.01). Pb concentrations ranged from 0.023 ± 0.011 mg/kg in Osijek–Baranja County to 0.031 ± 0.019 mg/kg in Međimurje County, and medians were similar in all regions (0.009–0.028 mg/kg), with moderate to high variability (CV = 49.8–100.4%). Differences in Pb concentrations among counties were not statistically significant (H = 2.96; *p* = 0.398), indicating a relatively homogeneous distribution of Pb in soils intended for maize cultivation.

### 3.3. Comparison of Metal Content Among Cereals

**Figure 2:**
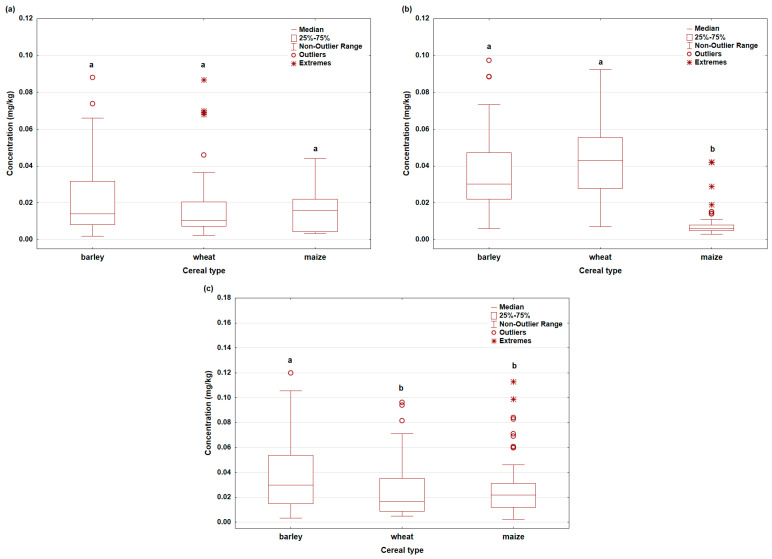
Concentrations of (**a**) arsenic (As), (**b**) cadmium (Cd), and (**c**) lead (Pb) in cereals from different regions of Croatia (mg/kg, dry weight). Boxes represent median and interquartile range (25th–75th percentile), whiskers indicate minimum and maximum values, and points denote outliers. Different lowercase letters above the boxes indicate statistically significant differences among cereals (Kruskal–Wallis test followed by post hoc multiple comparisons, *p* < 0.05).

Furthermore, the content of metals among barley, wheat, and maize was analyzed and is shown in Figure 2a–c, illustrating the distribution and differences in concentrations of As, Cd and Pb among cereals. Kruskal–Wallis analysis showed that As concentrations among cereals are not statistically significantly different (H = 4.40; *p* > 0.05). Although minor differences were noted in the mean ranks, they were not pronounced enough to be considered significant. Box-plot graphs additionally confirm the absence of significant differences, with partial overlapping of interquartile ranges, which may point to similar mechanisms of As uptake or limited bioavailability in the tested soils. For Cd, on the other hand, statistically significant differences were found among cereals (H = 124.47; *p* < 0.001). The lowest Cd concentrations were recorded in maize, while barley and wheat showed significantly higher values. Post hoc analysis confirmed that maize was statistically significantly different from barley and wheat (*p* < 0.05), while the differences between barley and wheat were not significant. Box-plot graphs clearly show lower medians and a narrower range of Cd concentrations in maize compared to other cereals. As for Pb, statistical analysis showed significant differences among cereals (H = 7.56; *p* < 0.05). The highest Pb concentrations were recorded in barley, while wheat and maize had lower and similar values. Post hoc test confirmed significant differences between barley and wheat and between barley and maize (*p* < 0.05), while the difference between wheat and maize was not significant. The graphic representation further illustrates that barley is characterized by a higher median and a wider range of Pb concentrations.

### 3.4. Transfer Factor (TF) of Metals in Cereals

**Figure 3:**
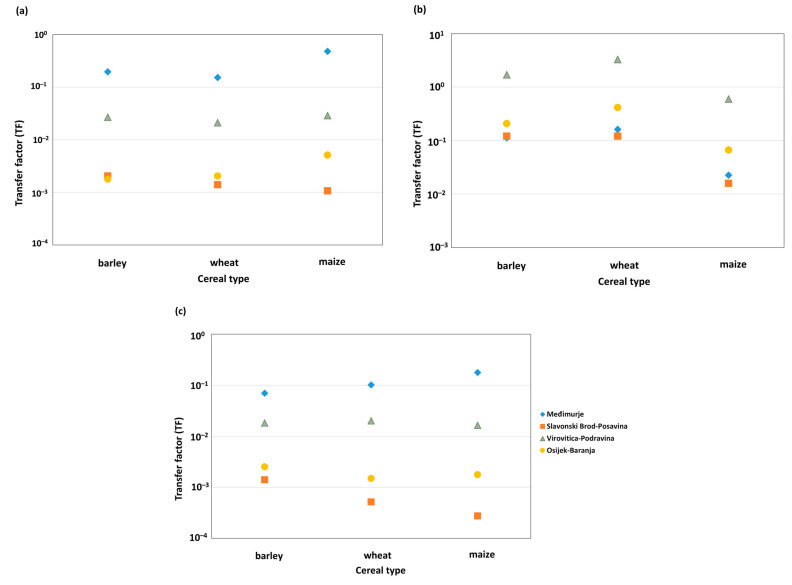
Soil-to-cereal transfer factors (TF) of (**a**) arsenic (As), (**b**) cadmium (Cd), and (**c**) lead (Pb) for barley, wheat, and maize across all investigated counties. TF values are based on median metal concentrations at the county level and are presented on a logarithmic scale. Soil and cereal samples were not paired at specific locations, so TF represents regional transfer patterns rather than site-specific variability.

TF values for As, Cd, and Pb showed significant spatial and species variability (Figure 3a–c). For As, the highest TF values were recorded in Međimurje, where maize had a TF of 0.478, indicating the highest accumulation of As in grain relative to soil. In the same county, TF values for barley (0.196) and wheat (0.152), were also higher than in other counties. In contrast, TF values in Slavonski Brod–Posavina were very low for all cereals (≈0.001–0.002), despite high soil As concentrations, suggesting low plant availability or efficient exclusion mechanisms. In Virovitica–Podravina and Osijek–Baranja counties, TF values for As were low (<0.03) but relatively consistent among cereals, with maize showing slightly higher TF values than barley and wheat. The Cd TF showed the largest range of values among metals. In Virovitica–Podravina County, Cd TF values for wheat (3.3) and barley (1.7) were the highest, while maize had a moderate TF value (0.6), indicating high bioavailability of Cd in the soil and a strong accumulation in grains, especially wheat. In Međimurje, TF values were moderate (wheat 0.162, barley 0.113, maize 0.023), whereas in Slavonski Brod–Posavina and Osijek–Baranja, TF values were generally low (<0.5), particularly in maize, reflecting limited transfer from soil to grain. For Pb, TF values in all counties, except Međimurje, were very low (<0.03), which confirms the limited mobility of Pb in soil and its poor transfer to cereals. In Međimurje, TF values for Pb were higher than in other counties, with maize having the highest TF (0.178), followed by wheat (0.102) and barley (0.070), suggesting increased bioavailability of Pb or specific conditions that favor its uptake into the plant, especially in maize.

Comparison of TF values between metals showed that Cd generally had the highest TF values, Pb the lowest, and As intermediate, with pronounced regional variability. Among cereals, maize had higher TF values for As and Pb in some counties, while for Cd, the highest values were often recorded in wheat and barley. Since TF represents the ratio of concentrations in the plant and soil, very low metal concentrations can result in high TF values. Consequently, these values do not necessarily reflect the absolute plant accumulation capacity.

### 3.5. Assessment of Non-Carcinogenic Risk for the Adult and Child Population (EDI, HQ and HI)

The assessment of the non-carcinogenic health risk for both the adult and child populations was based on the median concentrations of As, Cd, and Pb in barley, wheat and maize, aggregated across all studied counties. Since exposure concentrations in cereals are independent of age group, the same median values were applied for both adults and children, while differences in EDI and HQ resulted from differences in body weight assumptions. The calculated values of EDI, HQ, and total HI for adults and children are shown in Table 9 and Table 10.

For barley, the median concentrations were 0.012 mg/kg for As, 0.025 mg/kg for Cd, and 0.024 mg/kg for Pb (dry weight basis). The calculated EDI values resulted in very low HQ values for all observed metals in both populations (HQ < 0.03). Cd provided the highest contribution to the total hazard index (HQ_adults = 0.0099; HQ_children = 0.0208), followed by As (HQ_adults = 0.0027; HQ_children = 0.0058) and Pb (HQ_adults = 0.0023; HQ_children = 0.0049). The overall HI values were 0.015 for adults and 0.0314 for children, indicating a negligible non-carcinogenic risk.

Median concentrations of 0.010 mg/kg for As, 0.041 mg/kg for Cd, and 0.017 mg/kg for Pb were determined in wheat. Compared to other cereals, wheat showed the highest values of EDI and HQ for Cd, with HQ values of 0.244 for adults and 0.5125 for children. HQ for As and Pb were significantly lower (HQ_adults = 0.0339 and 0.0236; HQ_children = 0.0713 and 0.0495). Due to the dominant contribution of Cd, the overall HI for wheat was 0.3015 in adults and 0.6333 in children, which represents the highest HI value among the analyzed cereals, but still remained below the threshold value of 1. Median metal concentrations in maize were 0.018 mg/kg for As, 0.006 mg/kg for Cd, and 0.019 mg/kg for Pb. The calculated HQ values for all metals were low in both age groups, with As having the largest contribution to the total HI (HQ_adults = 0.0125; HQ_children = 0.0263), while the contributions of Cd and Pb were negligible (HQ < 0.01). The overall HI values were 0.0249 for adults and 0.0524 for children, indicating a low non-carcinogenic health risk.

**Figure 4:**
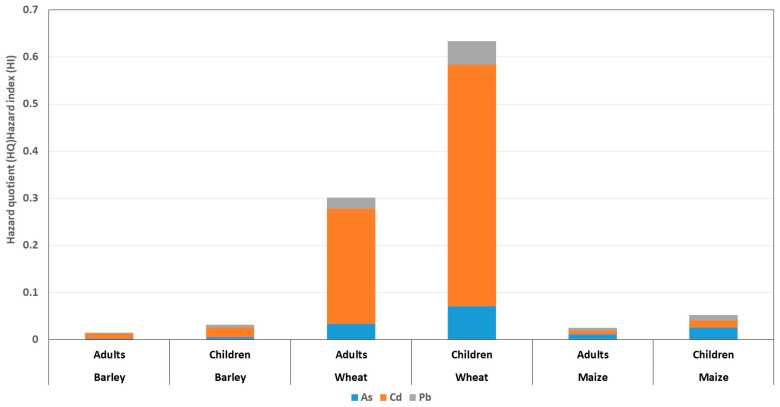
Contribution of arsenic (As), cadmium (Cd) and lead (Pb) to the total hazard index (HI) for barley, wheat and maize for adults and children, based on median metal concentrations.

The graphic representation (Figure 4) clearly highlights the differences in the structure of non-carcinogenic risk among the analyzed cereals and between the adult and child populations. The most pronounced risk was recorded for wheat, where Cd shows a dominant contribution to the HI. HQ values for Cd are significantly higher compared to those for As and Pb, especially in children, in whom HQ Cd exceeds 0.5. Although the total HI for wheat remains below the threshold value of 1, its relatively high value in the child population (0.6333) indicates that Cd is the key risk factor and potentially the most critical element in assessing the safety of this cereal. For barley, all individual HQ coefficients are low, with Cd making the largest, but still very limited, contribution to the total HI. The total HI values (0.015 for adults and 0.0314 for children) indicate a negligible health risk, without a pronounced dominance of any single metal. Maize shows a similar pattern of low HQ values, with As making a slightly larger contribution to total HI than Cd and Pb. However, the overall HI values remain very low (0.0249 for adults and 0.0524 for children), confirming that exposure through maize consumption does not represent a significant non-carcinogenic risk.

### 3.6. Probabilistic Risk Assessment Using Monte Carlo Simulation (EDI, HQ, and HI)

The results of the deterministic assessment were supplemented by a probabilistic analysis using Monte Carlo simulation, which accounts for the variability of metal concentrations in cereals and differences in daily food intake among population groups. The simulation was conducted for adult and child populations, using 10,000 iterations for each metal and total HI. The results are presented in Table 11 and Table 12, where for each metal and total HI, the P5 percentile, P50 (median), P95 percentile, and the proportion of iterations with HQ or HI greater than 1 (*p* > 1) are shown.

The results of the Monte Carlo simulation for barley show that all HQ coefficients are low and relatively homogeneous in both age segments. Cd makes the largest contribution to the total HI, but the HQ and HI values remain very low (HI_adults P50 = 0.0150; HI_children P50 = 0.0315), which confirms a negligible non-carcinogenic risk. The proportion of iterations with HQ or HI greater than 1 is 0%, which further confirms the safety of consumption. In wheat, the dominance of Cd is even more pronounced, especially in children, where the P95 of the total HI exceeds 1 (HI_children P95 = 1.0275) in about 6% of the simulations (*p* > 1 = 0.0601). As and Pb contribute significantly less to the total risk, and the median HI (P50 = 0.6336 for children) remains below the threshold value of 1, which indicates that wheat is relatively the most critical cereal among the analyzed cereals. In maize, all HQ coefficients and total HI are low in both age groups, with As contributing slightly more to the total HI than Cd and Pb, and the proportion of iterations with HQ or HI greater than 1 is 0%, which confirms the negligible non-carcinogenic risk from maize consumption.

**Figure 5:**
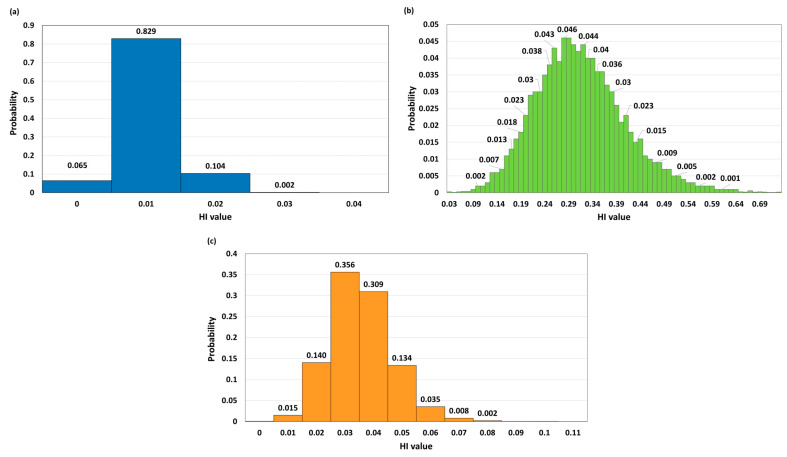
Distribution of the total hazard index (HI) for the adult population (70 kg) obtained by Monte Carlo simulation (10,000 iterations) for (**a**) barley, (**b**) wheat, and (**c**) maize. The histograms illustrate the total HI values based on the variability of As, Cd, and Pb concentrations in the grain.

**Figure 6:**
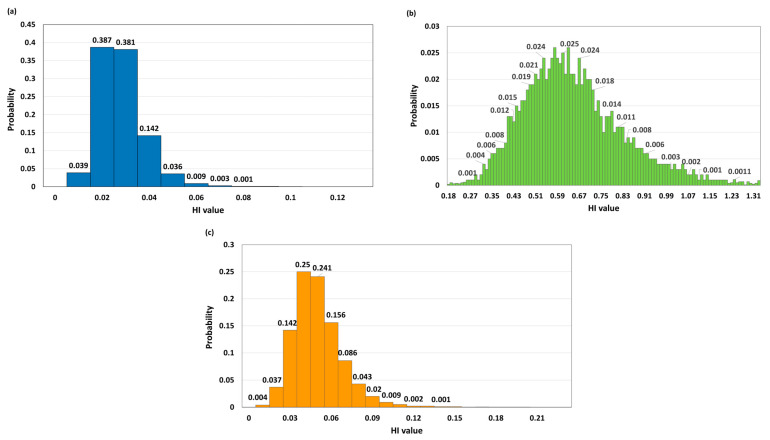
Distribution of the total hazard index (HI) for the child population (20 kg) obtained by Monte Carlo simulation (10,000 iterations) for (**a**) barley, (**b**) wheat and (**c**) maize. The histograms illustrate the total HI values based on the variability of As, Cd, and Pb concentrations in the grain.

Furthermore, the results of the Monte Carlo simulation are presented in histograms in Figure 5 for the adult population and in Figure 6 for the child population. The histograms show the distribution of the total HI for barley, wheat and maize. In adults (Figure 5a–c), the distribution of HI values for barley and maize is concentrated at very low values, with a narrow range and no iterations in which the HI exceeds 1, confirming a negligible non-carcinogenic risk. Wheat shows a wider distribution of HI values, but the median and P95 remain below the threshold value of 1, and the proportion of iterations with HI > 1 is 0%, indicating that even in the most unfavorable simulated scenarios, the safety threshold for the adult population is not exceeded.

In children (Figure 6a–c), the distributions are shifted towards higher HI values compared to adults, which is a consequence of lower body mass. For barley and maize, the HI remains low throughout the entire distribution range, without exceeding the value of 1. However, for wheat, in a smaller proportion of simulations (about 6%), the HI approaches or exceeds 1, confirming that wheat is the most critical cereal in assessing non-carcinogenic risk for the child population.

## 4. Discussion

### 4.1. Distribution of Metals in Soils of the Studied Counties

As, Cd and Pb concentrations in soils of the studied counties showed pronounced spatial heterogeneity, which is consistent with the known pedogenetic and geochemical characteristics of the Pannonian Plain [2]. The distribution of metals in agricultural soils is rarely homogeneous, even within relatively similar agroecological zones, as it is simultaneously influenced by natural geological factors, land use history, and long-term anthropogenic pressures [2,51,52]. Similar patterns of spatial variability of metals in soils have been recorded in numerous regional and European studies, which highlight that variability often transcends administrative boundaries and reflects local geochemical conditions [2,51,53,54].

In this study, the highest median concentrations of As and Pb were recorded in the Slavonski Brod–Posavina and Osijek–Baranja counties. Such a pattern can be explained by a combination of natural geogenic sources, related to the mineral composition of the parent substrate, and long-term anthropogenic loading. Eastern Croatia is historically an area of intensive agricultural production, but also transport and industrial activities, which have been recognized as important sources of Pb and Cd input into the soil, especially through atmospheric deposition and the application of phosphate fertilizers [2,51,52]. Pb, due to its low mobility and strong sorption on mineral and organic soil components, most often accumulates in surface horizons, while Cd shows greater mobility, especially in soils with lower pH values and lower organic matter content [2,55,56,57]. Despite the observed accumulation, median Pb concentrations in these counties remained below the upper soil guideline value of 300 mg/kg, while Cd concentrations were mostly within the 1–3 mg/kg guideline range and As concentrations were within 10–20 mg/kg [2,51].

The observed Cd concentrations in the soils of the studied counties are comparable to the values reported for other agricultural areas of Central and Eastern Europe, characterized by a moderate soil load with this element [2,51]. Although elevated values were recorded in some samples, they likely reflect a long-term accumulation process, rather than recent pollution, consistent with established behavior of Cd in agroecosystems [51,52,56,57]. On the other hand, relatively lower median metal concentrations in the soils of Međimurje County, despite pronounced local variability, can be associated with a different geological substrate, more favorable soil texture and lower impact of industrial pollution sources [2,53]. However, elevated coefficients of variation were also recorded in this region, indicating pronounced microlocational differences. Such spatial heterogeneity confirms that even areas perceived as less heavily burdened are not entirely homogeneous and that local conditions can have a significant impact on the accumulation of metals in soil [2,53,57,58]. Overall, the concentrations of As, Cd and Pb in soils remain below or within the recommended soil guideline values, indicating that the soils are generally suitable for agricultural use.

In the context of current national and European reference values for metals in soil, the obtained As, Cd and Pb concentrations are consistent with the soil guideline values. Despite regional differences and pronounced local variability, the soils of the studied counties do not show signs of pronounced contamination but rather reflect the combined influence of geogenic background and long-term, low-intensity anthropogenic loading [2,51,52]. In the Pannonian Plain, agriculture is predominantly rainfed, as climatic conditions are moderately warm and humid, with mean annual precipitation of roughly 550–900 mm and a concentration of rainfall in the growing season [33,34,59]. Under such conditions, crop production in the studied counties relies mainly on natural precipitation, while irrigation is applied only locally and on a limited share of high-value crops. Consequently, irrigation water is unlikely to represent a major and widespread source of As, Cd and Pb input into soils, and the observed spatial variability in metal concentrations is more plausibly driven by geogenic background, fertilizer inputs and atmospheric deposition [60,61,62].

These results highlight the importance of a regional approach in assessing soil metal contamination, as spatial variability can significantly influence the interpretation of risk and the assessment of potential metal transfer to plants [2,53,54]. By integrating soil guideline values, the study confirms that As, Cd, and Pb levels in these soils are unlikely to pose significant risks for agricultural use, underscoring the critical role of soil metal analysis in understanding the transfer of metals into the food chain [2,55,56,57].

### 4.2. Metal Concentrations in Cereals and Regional Patterns

Unlike in soil, As, Cd, and Pb concentrations in cereals showed significantly lower spatial variability among the studied counties. Such a pattern indicates that the total concentration of metals in the soil is not a direct indicator of their accumulation in edible parts of plants but that their bioavailability plays a crucial role, which depends on the physicochemical properties of the soil and on the physiological and biochemical mechanisms of the plant [63,64,65]. These findings align with a substantial body of European and international studies, documenting that even in areas with elevated concentrations of metals in the soil, their transfer to cereal grains remains restricted [25,63,66].

In most of the analyzed samples, the concentrations of As, Cd, and Pb in cereals were low and comparable to values recorded in other European and Mediterranean regions [26,67]. Regional differences in metal concentrations in grains were much less pronounced than in soils, indicating the efficiency of soil immobilization mechanisms, the selective barrier function of the root system, and restricted translocation of metals to the aerial parts of the plant [25,63,65]. In counties with higher soil metal concentrations, such as Slavonski Brod–Posavina and Osijek–Baranja, as well as those with lower median soil concentrations, like Međimurje, grain metal concentrations remained consistently low; this aligns with observations from mining and industrial zones elsewhere in Europe and Asia [25,63].

In the context of the current EU regulatory framework, Cd and Pb concentrations in all analyzed cereal samples were below the maximum permissible levels (MRLs) established for cereals intended for human consumption [26,67,68]. Specifically, Cd concentrations ranged below 0.10–0.20 mg/kg wet weight, and Pb concentrations remained well under 0.20 mg/kg wet weight, indicating that all samples are comfortably within the regulatory limits [41]. When expressed on a wet weight basis, median concentrations remained well below the maximum levels established by Commission Regulation (EU) 2023/915 [41]. Although differences were observed across counties and cereal types, none of the samples exceeded the legal thresholds, indicating the regulatory compliance of the analyzed products concerning Cd and Pb content.

For As, the EU currently regulates mainly inorganic As in rice, with no uniform limit values for total As in other cereals [68]. The observed total As concentrations in wheat, barley and maize were low (well below 10 mg/kg, as suggested in the literature for moderately contaminated soils), consistent with areas of moderate geogenic soil loading [25,68,69]. Therefore, the low levels and limited regional variability suggest minimal risk for human consumption.

Pb was present in very low concentrations in all analyzed cereals. Due to the strong sorption of Pb to soil particles and its limited mobility within the plant, Pb is predominantly retained in the root system, resulting is minimal translocation into the grain [67,70,71]. Therefore, spatial variations in Pb in the soil were not directly reflected within the cereal grain. The observed differences in metal concentrations among the analyzed cereal species indicate the importance of considering plant characteristics when interpreting the transfer of metals from the soil to edible parts of plants.

### 4.3. Differences in Metal Accumulation Among Cereal Species

A comparison of As, Cd, and Pb concentrations among barley, wheat, and maize has shown marked differences in metal accumulation patterns, confirming the important role of morphological and physiological properties of plants in regulating element uptake and distribution [25,72,73]. Such differences are often attributed to the root system architecture, growth dynamics, nutrient requirements and the specificity of transport and detoxification mechanisms, including the role of divalent cation transporters and vacuolar metal sequestration [66,73,74,75].

In this study, no significant differences in As concentrations were observed among cereals, suggesting similar mechanisms of As uptake or its limited bioavailability within the soils of the investigated counties [6,72]. The behavior of As in soil strongly depends on redox conditions, pH, and interactions with Fe and Al oxides; consequently under conditions of generally low mobility, observable variations among plant species may be less pronounced [6,69,72]. Similar patterns have been observed in other studies conducted within temperate agroecosystems, where As concentrations in grains of different cereals often do not show significant interspecific differences [6,72].

In contrast, Cd exhibited distinct variations in accumulation among cereals. Wheat and barley accumulated higher concentrations of Cd than maize, aligning with findings that wheat generally has a higher bioconcentration factor for Cd than maize [6,66]. This pattern is associated with higher efficiency of divalent cation transport systems and greater Cd translocation from roots to the aerial tissues in wheat and related cereals [66,74,75,76]. By contrast, maize is often described as a crop with a lower affinity for Cd accumulation in grain and more pronounced Cd retention in roots [6,25]. Differences in phenology and the longer growing season of winter cereals further prolong the window for potential Cd uptake compared to maize, potentially increasing the total accumulation in the grain [6,66,69].

For Pb, variations in concentrations among cereals were less pronounced, consistent with the well-documented low mobility of Pb and its strong sorption in the rhizosphere [71,72,77]. Lead is primarily retained in the root system, and its transfer to the aboveground parts of the plant and grain is generally limited, so interspecific differences in Pb in grains are often not statistically significant in soils without significant contamination [25,71,77].

Observed differences in metal accumulation among cereal species highlight the importance of quantifying soil-to-plant transfer specifically through bioconcentration factors or TF analysis to elucidate species-specific metal uptake and translocation strategies.

### 4.4. Transfer Factor (TF) and Metal Transfer from Soil to Plant

The TF has been used as a key indicator of the transfer of As, Cd, and Pb from soil to cereals, as it represents the ratio of metal concentrations in the plant and those in the corresponding soil. Although TF is often used in assessments of metal transfer in agroecosystems, its interpretation requires caution because it does not directly reflect the metal bioavailability but rather represents a simplified quantitative relationship between the two compartments [72,78]. The obtained TF values were interpreted according to the classification criteria defined in the Materials and Methods section. Elevated TF values may indicate higher metal bioavailability, specific physiological mechanisms of uptake and translocation within the plant, as well as the influence of soil properties, including chemical properties (e.g., pH, organic matter content, cation exchange properties, carbonate content) and physical properties (e.g., soil texture) can significantly affect metal mobility and availability [14,72,79].

The obtained TF values showed marked variability across counties, metals and cereal species, which is consistent with the known complexity of the processes regulating soil-to-plant metal transfer. Such variability results from the combined influence of soil properties, including chemical characteristics (pH, organic matter content, carbonate content and metal-binding phases) and physical characteristics (soil texture), as well as plant-specific mechanisms of metal uptake and regulation [6,7,66,72]. Therefore, TF does not only reflect the “strength” of transfer, but also the soil–plant interaction under specific agroecological conditions [7,66].

The relatively higher TF values recorded in counties with lower soil metal concentrations, most notably in Međimurje County, can be explained by the mathematical nature of the indicator itself. Since TF represents the ratio of plant metal concentration to soil metal concentration, low values in the denominator can result in elevated TF values, even when absolute plant metal concentrations remain low. For context, the TF values observed for Cd in wheat, maize, and barley were mostly within the typical ranges reported in literature (wheat: 0.16–0.42, maize: ≈0.10, barley: ≈0.24), indicating transfer within normal biological standards for these cereals. Deviations from these ranges may reflect specific soil characteristics, such as low pH or low organic matter, micronutrient deficiencies (e.g., Zn), or localized contamination [66,72].

By contrast, counties with higher soil metal concentrations exhibited lower TF values, potentially due to reduced bioavailability resulting from stronger metal sorption to mineral and organic soil components. Such behavior is particularly pronounced for Pb but also for As in soils rich in Fe oxides, where metal species are stably bound and become less bioavailable to plants [7,66,69,80]. Observed TF values for Pb were consistently low, generally within the typical range for cereals (0.0002–0.602). With wheat at approximately 0.0011, and barley slightly higher than wheat, maize demonstrated a strong exclusion capacity, restricting Pb translocation to 2–5% of soil content, confirming minimal transfer to grains even in soils with elevated Pb levels.

Differences in TF values between individual metals also reflect their specific chemical properties. Cd, due to its higher mobility and weaker sorption in soil, showed higher TF values compared to Pb, while TF values for As were moderate and strongly dependent on local soil conditions [6,7,66,69,75]. In contrast to the minimal TF values in wheat and barley (0.004–0.018 for wheat, 0.003–0.013 for barley), maize TF reached as high as 0.76 in localized high-contamination areas, aligning with the higher uptake potential reported for this crop under specific environmental conditions. These patterns confirm that Cd is generally the most mobile and readily transferred of the analyzed metals.

Although TF provides useful insight into the transfer of metals from soil to plants, its use as a stand-alone risk indicator is limited. Therefore, in this study, TF interpretation was combined with absolute metal concentrations in cereals and compared to literature-based typical TF ranges and EU maximum limits. Values within reference ranges indicate that metal transfer is within the normal biological standard for the crop, while elevated TF values may suggest soil-specific factors (pH, organic matter), nutrient deficiencies, or external contamination (especially for Pb). This approach allows for a more comprehensive assessment of potential risk [72,78,80].

### 4.5. Exposure and Non-Carcinogenic Health Risk Assessment

The dietary exposure assessment for As, Cd, and Pb was performed using a standard deterministic approach, commonly used in risk assessments related to dietary intake of contaminants. The calculation of the EDI was based on metal concentrations in cereals, average daily consumption, and reference body weight of the adult population, in accordance with the recommendations of international regulatory and scientific bodies (JECFA, USEPA, EFSA) [81,82]. Such an approach allows the comparability of the obtained results with previous studies conducted in different agroecological and nutritional contexts [82,83]. In addition to adults, children represent a particularly vulnerable group, as they consume more food per kilogram of body weight and exhibit higher dietary exposure and non-carcinogenic risk indices (HQ, HI) for heavy metals from cereals and cereal-based products in many studies [84,85,86].

The calculation of the HQ for individual metals is based on the ratio of the estimated daily intake to the corresponding reference dose (RfD), with the HQ providing an indicative measure of the potential non-carcinogenic risk [83,87]. In this study, the HQ values for As, Cd and Pb varied depending on the type of cereal and metal, which is consistent with the differences in their concentrations and accumulation patterns in the grain [83,88]. A similar range of HQ values has been reported in other European and Asian studies that assessed exposure to metals through the consumption of cereals and cereal products [88,89]. The total non-carcinogenic risk was estimated using the HI, which is the sum of the individual HQ values for the analyzed metals. The application of the HI allows considering the potential cumulative effect of multiple metals of similar toxicological profiles, which is particularly important in dietary exposure assessments where contaminants are ingested simultaneously [82,87,90]. In this context, the contribution of individual metals to the total HI differed among cereals, with the observed differences being associated with a higher proportion of Cd in wheat and a relatively lower contribution of Pb due to its low concentrations in the grain [83]. In the present study, although all calculated HI values for both adults and children remained below the critical threshold of 1, consistently higher values were observed in the child population. This difference was particularly marked for wheat, for which Cd represented the predominant contributor to the total HI. While the deterministic HI value for adults remained at a moderate level, the corresponding value for children approached approximately two-thirds of the established safety threshold, indicating a comparatively reduced margin of safety. The elevated exposure indices observed in children are primarily attributable to their lower body weight relative to cereal consumption, which results in a higher intake per kilogram of body mass. Although the HI did not surpass the reference value, these results underscore the necessity of explicitly considering sensitive subpopulations in dietary risk assessments, particularly in relation to staple foods such as wheat.

For As, the exposure assessment employed a conservative assumption that 50% of the total As is in the inorganic form, which is considered the most toxicologically relevant [88,91]. This approach is often used in situations where speciation analysis of As is not available and allows for reducing the likelihood of underestimating the potential risk [91]. Similar assumptions have been applied in other studies that assessed As exposure through cereals in the European context [83].

In addition to the deterministic assessment, the probabilistic Monte Carlo simulation enabled a more comprehensive characterization of exposure variability by integrating empirical distributions for body weight and cereal consumption. The simulation outcomes demonstrated that both the median (P50) and higher percentile exposure estimates remained below the critical hazard index (HI) threshold of 1 for adults and children, implying a low likelihood of non-carcinogenic effects under realistic exposure scenarios. However, the resulting exposure distributions consistently indicated higher values in children than in adults, corroborating their greater vulnerability. Consequently, the probabilistic framework enhances the robustness of the risk characterization and mitigates the uncertainty inherent in single-point deterministic estimates.

It is important to point out that the assessment of exposure in this research refers exclusively to the intake of metals through the consumption of the analyzed cereals, without including other food sources such as vegetables, fruits or drinking water. Since total metal exposure results from cumulative intake from different sources, the results of this assessment should be viewed as a partial component of total dietary exposure [82,88,92]. Such an approach is common in studies focused on specific food groups, but it carries certain limitations in the interpretation of the overall risk, especially for children, for whom multi-food and multi-metal exposure often leads to HI values above 1 [83,92].

## 5. Conclusions

This study assessed the concentrations of As, Cd, and Pb in soils and cereals from the Pannonian Plain of the Republic of Croatia in order to evaluate soil–plant transfer and the potential non-carcinogenic health risk for the adult and child population. The results revealed pronounced spatial variability of metal concentrations in soils among the studied counties, reflecting the combined influence of soil characteristics, geogenic background and long-term anthropogenic factors. However, despite locally elevated soil metal concentrations, As, Cd, and Pb levels in wheat, barley and maize grains were generally low and showed limited regional variability, indicating restricted metal bioavailability in the investigated agroecosystems.

Clear differences in metal accumulation were observed among cereal species, with wheat and barley exhibiting a higher tendency to accumulate Cd compared to maize, while differences in As and Pb concentrations among cereals were less pronounced. The values of the TF further demonstrated the complexity of soil–plant interactions and confirmed that total metal concentrations alone are not reliable predictors of metal accumulation in cereal grains, particularly in regions with low to moderate soil contamination. The exposure assessment indicated that the EDI, HQ, and total HI for all analyzed metals and cereal types remained below the corresponding reference values, suggesting a low non-carcinogenic health risk for the adult population under the assumptions applied in this study. Although higher exposure indices were identified in children as a consequence of their lower body weight, all calculated HI values remained below the critical threshold of 1. Furthermore, the probabilistic Monte Carlo simulation corroborated the robustness of the deterministic risk assessment and demonstrated a low probability of exceeding the reference risk values under realistic exposure scenarios. All reported concentrations and risk assessments were expressed on a wet weight basis, remaining well below the maximum levels established by Commission Regulation (EU) 2023/915, confirming the safety of the analyzed cereals for human consumption.

Overall, the findings indicate that the consumption of the analyzed cereals from the studied areas does not pose a significant health risk and highlight the importance of integrated soil–plant monitoring approaches that consider both soil contamination levels and soil-to-crop transfer processes to support food safety and public health protection.

## Figures and Tables

**Table 1:** Distribution of collected cereal samples by counties and type (2019–2020, n = number of samples).

County (R)	Wheat (n)	Barley (n)	Maize (n)	Total Samples
Međimurje (R1)	5	10	15	30
Slavonski Brod–Posavina (R2)	24	26	33	83
Virovitica–Podravina (R3)	17	9	19	45
Osijek–Baranja (R4)	11	5	22	38
Total	57	50	89	196

**Table 2:** Analyzed isotopes of metallic elements, corresponding internal standards, and collision-reaction cell parameters (He, KED mode).

Element	Isotope Analyzed (*m*/*z*)	Internal Standard	Collision-Reaction Mode	Remark
As	75	Rh-103	He (KED)	Reduction ^40^Ar^35^C
Cd	111	Rh-103	He (KED)	Alternative: ^114^Cd
Pb	208	Rh-103	He (KED)	Highest sensitivity

**Table 3:** Limits of detection (LOD) and quantification (LOQ) for arsenic (As), cadmium (Cd), and lead (Pb) in grain and soil samples (mg/kg dry matter).

Element	LOD (mg/kg)	LOQ (mg/kg)
As	0.010	0.025
Cd	0.004	0.01
Pb	0.002	0.05

**Table 4:** Regulatory guidelines and maximum levels for arsenic (As), cadmium (Cd) and lead (Pb) in agricultural soils and cereals.

Element	Soil Guideline Value (mg/kg)	Maximum Level in Cereal (mg/kg Wet Weight)	Regulatory Source
As	10–20 [2]	No uniform total As maximum for cereals; inorganic As regulated in specific products	EU 2023/915 [38]
Cd	1–3 [2]	0.10–0.20 (depending on cereal type)	EU 2023/915 [38]
Pb	50–300 [2]	0.20	EU 2023/915 [41]

**Table 5:** Descriptive statistics of arsenic (As), cadmium (Cd), and lead (Pb) concentrations in soils from different regions of Croatia (mg/kg, dry weight).

County/Statistical Parameters	As	Cd	Pb
Međimurje (n = 8)			
Mean ± SD	0.050 ± 0.015	0.222 ± 0.058	0.154 ± 0.037
Median	0.046 ^a^	0.222 ^b^	0.157 ^a^
Min–Max	0.034–0.074	0.124–0.303	0.100–0.224
CV (%)	30.18	25.89	24.01
Slavonski Brod–Posavina (n = 18)			
Mean ± SD	11.811 ± 4.991	0.368 ± 0.172	31.911 ± 7.735
Median	12.170 ^c^	0.375 ^b,c^	32.755 ^b,c^
Min–Max	3.240–21.390	0.050–0.660	21.600–50.160
CV (%)	42.26	46.86	24.24
Virovitica-Podravina (n = 8)			
Mean ± SD	0.499 ± 0.075	0.011 ± 0.004	1.669 ± 0.599
Median	0.520 ^a,b^	0.010 ^a^	1.500 ^a,b^
Min–Max	0.330–0.580	0.010–0.020	1.090–2.700
CV (%)	15.10	31.43	35.88
Osijek–Baranja (n = 9)			
Mean ± SD	3.362 ± 1.557	0.103 ± 0.035	11.224 ± 5.041
Median	3.910 ^b,c^	0.120 ^a,b^	10.700 ^b,c^
Min–Max	1.360–4.900	0.050–0.140	6.460–23.100
CV (%)	46.32	33.52	44.91
Soil guideline value (Table 4)	10–20	1–3	50–300

Different superscript letters within the same column indicate statistically significant differences among regions (Kruskal–Wallis test followed by post hoc multiple comparisons, *p* < 0.05).

**Table 6:** Descriptive statistics of arsenic (As), cadmium (Cd), and lead (Pb) concentrations in barley from different regions of Croatia (mg/kg, dry weight).

County/Statistical Parameters	As	Cd	Pb
Međimurje (n = 10)			
Mean ± SD	0.014 ± 0.014	0.027 ± 0.012	0.022 ± 0.017
Median	0.009 ^a,b^	0.025 ^a,b^	0.011 ^a^
Min–Max	0.003–0.048	0.006–0.047	0.006–0.051
CV (%)	100.91	43.09	78.78
Slavonski Brod–Posavina (n = 26)			
Mean ± SD	0.030 ± 0.022	0.049 ± 0.022	0.051 ± 0.034
Median	0.025 ^b^	0.046 ^b^	0.046 ^b^
Min–Max	0.007–0.088	0.017–0.097	0.009–0.120
CV (%)	72.91	45.81	67.33
Virovitica–Podravina (n = 9)			
Mean ± SD	0.018 ± 0.014	0.018 ± 0.008	0.026 ± 0.016
Median	0.014 ^a,b^	0.017 ^a^	0.020 ^a,b^
Min–Max	0.002–0.040	0.007– 0.030	0.003–0.054
CV (%)	78.45	45.64	62.93
Osijek–Baranja (n = 5)			
Mean ± SD	0.007 ± 0.002	0.026 ± 0.007	0.028 ± 0.005
Median	0.007 ^a^	0.025 ^a,b^	0.027 ^a,b^
Min–Max	0.004–0.008	0.018–0.037	0.022–0.033
CV (%)	32.59	27.31	17.61
Maximum level (Table 4)	– *	0.12–0.23 **	0.23 **

* No uniform maximum level is established for total As in cereals. ** Maximum levels for Cd and Pb in cereals (wet weight basis) were converted to dry weight, assuming 14% moisture content, to allow direct comparison with analytical results. Different superscript letters within the same column indicate statistically significant differences among regions (Kruskal–Wallis test followed by post hoc multiple comparisons, *p* < 0.05).

**Table 7:** Descriptive statistics of arsenic (As), cadmium (Cd), and lead (Pb) concentrations in wheat from different regions of Croatia (mg/kg, dry weight).

County/Statistical Parameters	As	Cd	Pb
Međimurje (n = 5)			
Mean ± SD	0.007 ± 0.003	0.030 ± 0.013	0.015 ± 0.009
Median	0.007 ^a^	0.036 ^a^	0.016 ^a^
Min–Max	0.002–0.009	0.014–0.042	0.005–0.028
CV (%)	41.71	44.80	59.56
Slavonski Brod–Posavina (n = 24)			
Mean ± SD	0.025 ± 0.023	0.050 ± 0.028	0.030 ± 0.028
Median	0.017 ^b^	0.046 ^a^	0.017 ^a^
Min–Max	0.006–0.087	0.007–0.092	0.007–0.096
CV (%)	94.51	55.97	91.87
Virovitica–Podravina (n = 17)			
Mean ± SD	0.014 ± 0.010	0.040 ± 0.019	0.024 ± 0.016
Median	0.011 ^a,b^	0.033 ^a^	0.022 ^a^
Min–Max	0.003–0.036	0.013–0.083	0.006–0.057
CV (%)	70.15	48.39	69.18
Osijek–Baranja (n = 11)			
Mean ± SD	0.011 ± 0.012	0.041 ± 0.017	0.024 ± 0.018
Median	0.008 ^a^	0.05 ^a^	0.016 ^a^
Min–Max	0.004–0.046	0.011–0.061	0.008–0.071
CV (%)	108.99	41.33	75.94
Maximum level (Table 4)	– *	0.12–0.23 **	0.23 **

* No uniform maximum level is established for total As in cereals. ** Maximum levels for Cd and Pb in cereals (wet weight basis) were converted to dry weight, assuming 14% moisture content, to allow direct comparison with analytical results. Different superscript letters within the same column indicate statistically significant differences among regions (Kruskal–Wallis test followed by post hoc multiple comparisons, *p* < 0.05).

**Table 8:** Descriptive statistics of arsenic (As), cadmium (Cd), and lead (Pb) concentrations in maize from different regions of Croatia (mg/kg, dry weight).

County/Statistical Parameters	As	Cd	Pb
Međimurje (n = 15)			
Mean ± SD	0.019 ± 0.011	0.006 ± 0.003	0.031 ± 0.019
Median	0.022 ^a^	0.005 ^a^	0.028 ^a^
Min–Max	0.004–0.044	0.004–0.015	0.005–0.084
CV (%)	55.92	53.60	61.60
Slavonski Brod–Posavina (n = 33)			
Mean ± SD	0.014 ± 0.009	0.008 ± 0.008	0.027 ± 0.026
Median	0.013 ^a^	0.006 ^a^	0.009 ^a^
Min–Max	0.004–0.032	0.003–0.042	0.005–0.113
CV (%)	69.73	98.02	100.39
Virovitica–Podravina (n = 19)			
Mean ± SD	0.011 ± 0.007	0.007 ± 0.002	0.025 ± 0.020
Median	0.015 ^a^	0.006 ^a,b^	0.018 ^a^
Min–Max	0.004–0.023	0.005–0.011	0.002–0.099
CV (%)	64.41	24.01	82.92
Osijek–Baranja (n = 22)			
Mean ± SD	0.018 ± 0.013	0.009 ± 0.007	0.023 ± 0.011
Median	0.020 ^a^	0.008 ^b^	0.019 ^a^
Min–Max	0.004–0.038	0.006–0.042	0.009–0.060
CV (%)	70.31	78.48	49.75
Maximum level (Table 4)	– *	0.12–0.23 **	0.23 **

* No uniform maximum level is established for total As in cereals. ** Maximum levels for Cd and Pb in cereals (wet weight basis) were converted to dry weight, assuming 14% moisture content to allow direct comparison with analytical results. Different superscript letters within the same column indicate statistically significant differences among regions (Kruskal–Wallis test followed by post hoc multiple comparisons, *p* < 0.05).

**Table 9:** Estimated daily intake (EDI), hazard quotient (HQ) and hazard index (HI) for arsenic (As), cadmium (Cd), and lead (Pb) in barley, wheat, and maize, calculated for the adult population (body weight = 70 kg) based on median metal concentrations.

Cereal	Metal	Median (mg/kg)	EDI (mg/kg day)	HQ	HI
Barley	As	0.012 (0.010)	8.21 × 10^−7^	0.0027	0.0150
	Cd	0.025 (0.022)	3.57 × 10^−6^	0.0099	
	Pb	0.024 (0.021)	3.47 × 10^−6^	0.0023	
Wheat	As	0.010 (0.009)	1.02 × 10^−5^	0.0339	0.3015
	Cd	0.041 (0.035)	8.79 × 10^−5^	0.2440	
	Pb	0.017 (0.015)	3.54 × 10^−5^	0.0236	
Maize	As	0.018 (0.015)	3.75 × 10^−6^	0.0125	0.0249
	Cd	0.006 (0.005)	2.57 × 10^−6^	0.0071	
	Pb	0.019 (0.016)	7.93 × 10^−6^	0.0053	

Median metal concentrations are expressed on a dry weight basis. Corresponding wet weight concentrations (shown in parentheses) were estimated assuming an average grain moisture content of 14% for comparison with regulatory maximum levels. EDI, HQ and HI were calculated using dry weight concentrations, representing a conservative exposure assessment. EDI was calculated based on the median concentrations of metals in cereals. For As, it was assumed that the inorganic form constitutes 50% of the total content. HQ = EDI/reference value (TDI or BMDL, depending on the metal). HI represents the sum of HQ values. HQ and HI < 1 indicate negligible non-carcinogenic risk.

**Table 10:** Estimated daily intake (EDI), hazard quotient (HQ) and hazard index (HI) for arsenic (As), cadmium (Cd), and lead (Pb) in barley, wheat, and maize, calculated for the adult population (body weight = 20 kg) based on median metal concentrations.

Cereal	Metal	Median (mg/kg)	EDI (mg/kg day)	HQ	HI
Barley	As	0.012 (0.010)	1.73 × 10^−6^	0.0058	0.0314
	Cd	0.025 (0.022)	7.50 × 10^−6^	0.0208	
	Pb	0.024 (0.021)	7.29 × 10^−6^	0.0049	
Wheat	As	0.010 (0.009)	2.14 × 10^−5^	0.0713	0.6333
	Cd	0.041 (0.035)	1.85 × 10^−4^	0.5125	
	Pb	0.017 (0.015)	7.43 × 10^−5^	0.0495	
Maize	As	0.018 (0.015)	7.86 × 10^−6^	0.0263	0.0524
	Cd	0.006 (0.005)	5.40 × 10^−6^	0.0150	
	Pb	0.019 (0.016)	1.67 × 10^−5^	0.0111	

Median metal concentrations are expressed on a dry weight basis. Corresponding wet weight concentrations (shown in parentheses) were estimated assuming an average grain moisture content of 14% for comparison with regulatory maximum levels. EDI, HQ and HI were calculated using dry weight concentrations, representing a conservative exposure assessment. EDI was calculated based on the median concentrations of metals in cereals. For As, it was assumed that the inorganic form constitutes 50% of the total content. HQ = EDI/reference value (TDI or BMDL, depending on the metal). HI represents the sum of HQ values. HQ and HI < 1 indicate negligible non-carcinogenic risk.

**Table 11:** Monte Carlo simulation: hazard quotient (HQ) and hazard index (HI) for the adult population (70 kg).

Cereal	Metal	HQ (P5)	HQ (P50)	HQ (P95)	*p* > 1
Barley	As	0.0018	0.0027	0.0040	0
	Cd	0.0064	0.0099	0.0145	0
	Pb	0.0015	0.0023	0.0034	0
	HI	0.0096	0.0150	0.0220	0
Wheat	As	0.0192	0.0340	0.0519	0
	Cd	0.1380	0.2449	0.3736	0
	Pb	0.0133	0.0237	0.0361	0
	HI	0.1705	0.3026	0.4616	0
Maize	As	0.0077	0.0125	0.0188	0
	Cd	0.0044	0.0071	0.0107	0
	Pb	0.0032	0.0053	0.0079	0
	HI	0.0153	0.0249	0.0374	0

Results of a Monte Carlo simulation with 10,000 iterations; P5, P50 (median), and P95 percentiles for HQ and HI are shown; *p* > 1 indicates the proportion of iterations with values greater than 1.

**Table 12:** Monte Carlo simulation: hazard quotient (HQ) and hazard index (HI) for the child population (20 kg).

Cereal	Metal	HQ (P5)	HQ (P50)	HQ (P95)	*p* > 1
Barley	As	0.0037	0.0058	0.0091	0
	Cd	0.0134	0.0209	0.0330	0
	Pb	0.0031	0.0049	0.0077	0
	HI	0.0203	0.0315	0.0497	0
Wheat	As	0.0439	0.0713	0.1156	0
	Cd	0.3159	0.5128	0.8315	0.0134
	Pb	0.0305	0.0495	0.0803	0
	HI	0.39032	0.6336	1.0275	0.0601
Maize	As	0.0166	0.0262	0.0421	0
	Cd	0.0095	0.0150	0.0241	0
	Pb	0.0070	0.0111	0.0178	0
	HI	0.0330	0.0523	0.0840	0

Results of a Monte Carlo simulation with 10,000 iterations; P5, P50 (median), and P95 percentiles for HQ and HI are shown; *p* > 1 indicates the proportion of iterations with values greater than 1.

## Data Availability

The original contributions presented in the study are included in the article. Further inquiries can be directed to the corresponding authors.

## Abbreviations

The following abbreviations are used in this manuscript:

As	Arsenic
BMDL	Benchmark Dose Lower Confidence Limit
BW	Body Weight
Cd	Cadmium
CV	Coefficient of Variation
EDI	Estimated Daily Intake
EFSA	European Food Safety Authority
EU	European Union
H	Kruskal–Wallis test statistic
HI	Hazard Index
HQ	Hazard Quotient
ICP-MS	Inductively Coupled Plasma Mass Spectrometry
IR	Ingestion Rate
KED	Kinetic Energy Discrimination
LOD	Limit of Detection
LOQ	Limit of Quantification
Pb	Lead
QA/QC	Quality Assurance/Quality Control
RSD	Relative Standard Deviation
SD	Standard Deviation
TF	Transfer Factor
TDI	Tolerable Daily Intake
TWI	Tolerable Weekly Intake
